# Inflammatory pseudotumor of the Kidney

**DOI:** 10.1186/1477-7819-5-106

**Published:** 2007-09-24

**Authors:** David R Selvan, Joe Philip, Ramaswamy Manikandan, Timothy R Helliwell, Gabriel HR Lamb, Anthony D Desmond

**Affiliations:** 1 Department of Urology, Royal Liverpool University Hospital, Liverpool, UK; 2 Department of Pathology, Royal Liverpool University Hospital, Liverpool, UK; 3 Department of Radiology, Royal Liverpool University Hospital, Liverpool, UK

## Abstract

**Background:**

Inflammatory pseudotumor of the kidney or inflammatory myofibroblastic tumor (IMT) is composed of spindle cells admixed with variable amount of proliferating myofibroblasts, fibroblasts, extracellular collagen, lymphocytes and plasma cells. This mainly affects the urinary bladder or prostate. Renal involvement is rare.

**Case presentation:**

A 56 year-old man was diagnosed with asymptomatic left sided hydronephrosis while being investigated for rheumatoid arthritis. CT scan imaging showed ill defined fascial plains around the kidney and thickening around the renal hilum suggestive of localized inflammatory change. Worsening intermittent left loin pain with increasing hydronephrosis, significant cortical thinning and marked deterioration of renal function necessitated nephrectomy. Macroscopy showed a hydronephrotic fibrotic kidney with microscopy and immunohistochemistry consistent with a histological diagnosis of IMT.

**Conclusion:**

We report a case of an inflammatory pseudotumor of the kidney. It is unique in that the patient presented with painless hydronephrosis followed two years later with progressive deterioration in renal function and worsening loin pain.

## Background

Inflammatory myofibroblastic tumor (IMT) or pseudotumor is a rare benign tumor that can be seen in various organs. It is of unknown etiology and difficult to differentiate from malignancy. Histologically, there is evidence of marked proliferation of myofibroblasts, fibroblasts, histiocytes, and plasma cells. There have been no reports of recurrence.

We report a unique case of inflammatory pseudotumor of the kidney; the patient presented with painless hydronephrosis followed two years later with progressive deterioration in renal function and worsening loin pain.

## Case presentation

**Figure 1:**
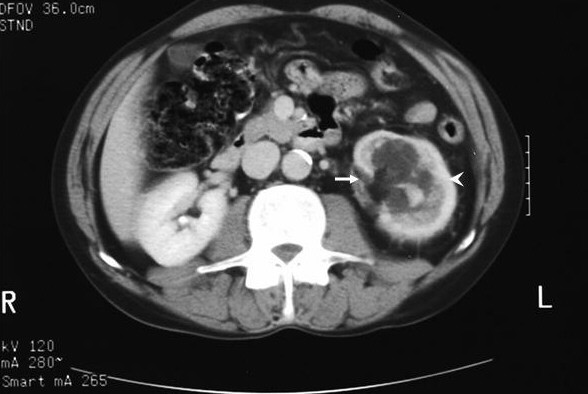
CT scan showing PUJ obstruction (arrow) with ill defined fascial planes around the left kidney (arrow head) that is consistent with inflammation.

A 56 year-old man was diagnosed with asymptomatic left sided hydronephrosis while being investigated for rheumatoid arthritis. Investigations included ultrasonography, intravenous urography, retrograde uretero-pyelography, Computed tomography (CT) imaging, renography and screening for tuberculosis. Radiological appearances were not typical of classical pelvi-ureteric junction obstruction with normal funneling and ready contrast drainage into upper ureter. Isotope renography revealed a 25% split left kidney function with a poor response to frusemide diuresis. CT scan imaging showed ill defined fascial plains around the kidney and thickening around the renal hilum suggestive of localized inflammatory change (Figure 1). No surgical intervention was undertaken as the patient was asymptomatic with significant ischaemic heart disease. Two years later, he developed worsening intermittent left loin pain with increasing hydronephrosis, significant cortical thinning and marked deterioration of renal function. Following coronary artery bypass surgery, he opted for a left nephrectomy. Patient remains pain-free at one year follow-up.

**Figure 2:**
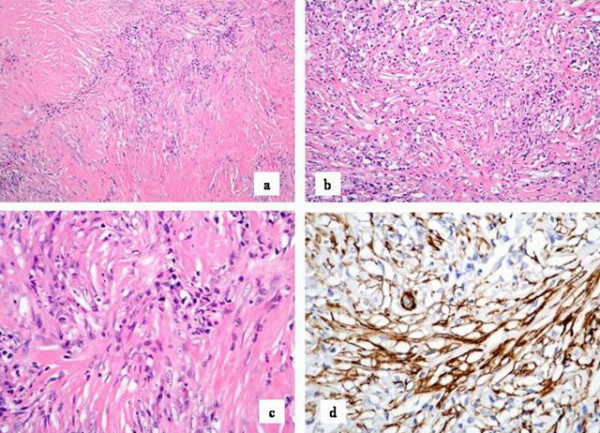
Photomicrograph showing a] Vaguely nodular areas of collagenous tissue with scattered spindle cells and darker inflammatory cells at the edges of the collagenous nodules. (H & E, ×60). b] More cellular area where there is a pale, eosinophilic collagenous background with pale spindle cells and darker inflammatory cells scattered without any particular pattern. (H & E, 150). c] Pale nuclei of the spindle cells with scattered darker lymphocytes and plasma cells in a pale, eosinophilic background. (H & E, ×250). d] Immunocytochemical labeling for smooth muscle actin, shown as brown strands of filamentous material in the cytoplasm of the myofibroblasts. (Immunocytochemical labeling, smooth muscle actin, ×250).

Macroscopy showed a hydronephrotic kidney with areas of fibrosis adjacent to the renal pelvis and peri-renal fat. Microscopically, within the area of fibrosis, there were hypocellular interweaving thick collagen bundles with extensive hyalination intermingled with bland spindle cells. Inflammatory cells including lymphocytes, eosinophils and plasma cells were also found (Figure 2a–c). There were some irregular projections of this fibrous tissue into the adjacent fat.

**Figure 3:**
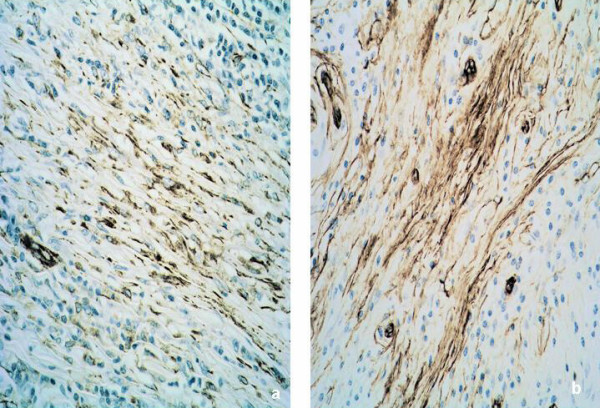
Immunocytochemical labeling for vimentin (3a) and CD34 (3b) showing spindle cells within the tumour positive for vimentin and CD 34. (×250)

Immunohistochemistry showed positivity for smooth muscle actin (Figure 2d), vimentin and CD34 (Figure 3a &3b) but negativity for ALK-1 consistent with a histological diagnosis of IMT.

## Discussion

Inflammatory pseudotumour of the kidney or IMT is a disease of young people and unknown etiology, affecting females more than males [1]. Differential diagnoses include malignant tumors such as renal cell carcinoma, sarcomatoid renal cell carcinoma, inflammatory fibrosarcoma, malignant fibrous histiocytoma, low grade neurogenic tumor, myxoid leimyosarcoma [2] and non-malignant tumors such as angiomyolipoma, xanthogranuloma pyelonephritis and plasma cell granuloma [3]. Patients usually present with haematuria and/or abdominal pain. Clinical examination and radiological investigations are often inconclusive. It is difficult to obtain enough tissue by CT-guided fine needle aspiration to make a definitive histological diagnosis [3,4] and most diagnoses have been made at the time of surgical intervention.

Histologically, they consist of proliferation of spindle cells admixed with various amounts of lymphocyplasmacytic infiltrate with consistent expression of vimentin and smooth muscle actin [5,6]. These tumors are strongly positive for CD 34 reactivity. ALK is only positive in 50% and it generally tends to be positive in younger patients [7]. The architectural appearances vary and have been described as a patternless pattern [2].

IMT generally tend to lack severe cytologic atypia and less mitosis than sarcomas and also generally tend to be negative for p53 which is positive in sarcomas [2].

Local recurrences and malignant transformation have been reported in a small subset of patients, but these generally tend to be where complete resection has been impossible [1,8,9].

We report a rare case of an inflammatory pseudotumour of the kidney. It is unique in that the patient presented with painless hydronephrosis and progressive deterioration in renal function while developing worsening loin pain. We suspect that the inflammatory reaction could have occurred as a consequence of chronic pelvi-ureteric junction obstruction.

## Conclusion

We report a rare case of an inflammatory pseudotumor of the kidney. It is unique in that the patient presented with painless hydronephrosis and progressive deterioration in renal function while developing worsening loin pain. We suspect that the inflammatory reaction could have occurred as a consequence of chronic pelvi-ureteric junction obstruction.

## Competing interests

The author(s) declare that they have no competing interests.

## Authors' contributions

**JP, DRS **and **RM **conceptualized the case report, conducted the literature search. **DRS**, **JP**, **RM **and **ADD **were involved in direct clinical management of the patient. **GHRL **conducted the radiological investigations and **TRH **carried out the pathological analysis and immunohistochemistry. All authors helped to draft the manuscript. All authors read and approved the final manuscript.
